# The Ability of Postimmunobiotics from *L. rhamnosus* CRL1505 to Protect against Respiratory Syncytial Virus and Pneumococcal Super-Infection Is a Strain-Dependent Characteristic

**DOI:** 10.3390/microorganisms10112185

**Published:** 2022-11-03

**Authors:** Fernanda Raya Tonetti, Patricia Clua, Kohtaro Fukuyama, Guillermo Marcial, Jacinto Sacur, Gabriela Marranzino, Mikado Tomokiyo, Guadalupe Vizoso-Pinto, Apolinaria Garcia-Cancino, Shoichiro Kurata, Haruki Kitazawa, Julio Villena

**Affiliations:** 1 Laboratory of Immunobiotechnology, Reference Centre for Lactobacilli (CERELA-CONICET), San Miguel de Tucuman 4000, Argentina; 2 Laboratory of Animal Food Function, Food and Feed Immunology Group, Graduate School of Agricultural Science, Tohoku University, Sendai 981-8555, Japan; 3 Livestock Immunology Unit, International Education and Research Center for Food and Agricultural Immunology (CFAI), Graduate School of Agricultural Science, Tohoku University, Sendai 981-8555, Japan; 4 Infection Biology Laboratory, Instituto Superior de Investigaciones Biológicas (INSIBIO), CONICET-UNT, San Miguel de Tucuman 4000, Argentina; 5 Facultad de Ciencias de la Salud, Universidad del Norte Santo Tomás de Aquino (UNSTA), San Miguel de Tucuman 4000, Argentina; 6 Laboratory of Bacterial Pathogenicity, Faculty of Biological Sciences, University of Concepcion, Concepcion 4030000, Chile; 7 Laboratory of Molecular Genetics, Graduate School of Pharmaceutical Sciences, Tohoku University, Sendai 980-8578, Japan

**Keywords:** *Lacticaseibacillus rhamnosus* CRL1505, peptidoglycan, respiratory syncytial virus, respiratory superinfection, *Streptococcus pneumoniae*, TLR3, antiviral immunity

## Abstract

Previously, we demonstrated that the non-viable strain *Lacticaseibacillus rhamnosus* CRL1505 (NV1505) or its purified peptidoglycan (PG1505) differentially modulated the respiratory innate antiviral immune response triggered by Toll-like receptor (TLR)-3 activation in infant mice, improving the resistance to primary respiratory syncytial virus (RSV) infection and secondary pneumococcal pneumonia. In this work, we evaluated the effect of other non-viable *L. rhamnosus* strains and their peptidoglycans on the respiratory immune response and their impact on primary and secondary respiratory infections. In addition, the duration of the protective effect induced by NV1505 and PG1505 as well as their ability to protect against different *Streptococcus pneumoniae* serotypes were evaluated. Our results showed that among the five selected *L. rhamnosus* strains (CRL1505, CRL498, CRL576, UCO25A and IBL027), NV1505 and NVIBL027 improved the protection against viral and pneumococcal infections by modulating the respiratory immune response. Of note, only the PG1505 presented immunomodulatory activities when compared with the other purified peptidoglycans. Studies on alveolar macrophages showed that NV1505 and PG1505 differentially modulated the expression of *IL-6*, *IFN-γ*, *IFN-β*, *TNF-α*, *OAS1*, *RNAseL* and *IL-27* genes in response to RSV infection, and *IL-6*, *IFN-γ*, *IL-1β*, *TNF-α*, *CCL2*, *CXCL2, CXCL10* and *IL-27* in response to pneumococcal challenge. Furthermore, we demonstrated that NV1505 and PG1505 treatments protected mice against secondary pneumococcal pneumonia produced by different serotypes of *S. pneumoniae* until 30 days after stimulation with poly(I:C). This work advances the characterization of the protective effect of NV1505 and PG1505 by demonstrating that they increase resistance against the pneumococcal serotypes 3, 6B, 14 and 19F, with an effect that lasts up to 30 days after the primary viral inflammation. The results also confirm that the immunomodulatory properties of NV1505 and PG1505 are unique and are not shared by other members of this species, and suggest the existence of a capacity to stimulate trained immunity in alveolar macrophages.

## 1. Introduction

Respiratory infections are of major importance in global health because of their capacity to rapidly spread among countries and cause of a high degree of morbidity/mortality in high-risk populations. The infections caused by *Streptococcus pneumoniae* and by respiratory syncytial virus (RSV) are epidemiologically the most important and aggressive in children [1,2]. RSV can spread to the lower respiratory tract in susceptible individuals, causing severe symptoms associated with cytopathic abnormalities. It can also cause dysregulation of the immune response, resulting in lung tissue injury, loss of function and even death [2]. In addition, RSV is responsible for an increment in the frequency [3] and severity [4] of pneumococcal disease. Indeed, the lethal combination of primary viral infections with secondary bacterial pneumonia is responsible of the high mortality reported for respiratory infections in children [5,6]. The virus-mediated destruction of the respiratory epithelium, the impairment of the mucociliary barrier defenses [7], the up-regulation of epithelial [8] and viral [9] proteins that promote adhesion and the interaction of the RSV G protein with the pneumococcal penicillin-binding protein 1a that increases bacterial virulence [7] are responsible for the greater severity of secondary pneumococcal pneumonia compared to the primary infection. Therefore, strategies aimed at preventing primary viral infections or reducing their severity could affect the morbidity and mortality of secondary bacterial infections like that produced by the RSV and *S. pneumoniae* interaction.

In recent years, probiotic bacteria with the ability to modulate the mucosal immune system (immunobiotics) have been proposed as a promising alternative for the prevention of viral and bacterial respiratory infections [10,11,12,13,14]. In this regard, studies reported that the priming of the respiratory tract with live or heat-inactivated *Lactiplantibacillus plantarum* resulted in robust and sustained protection against a subsequent lethal respiratory virus infection in association with remarkable inflammatory suppression [15,16,17]. Similarly, we have demonstrated previously that the nasal priming of infant mice with non-viable *Lacticaseibacillus rhamnosus* CRL1505 (NV1505) or its purified peptidoglycan (PG1505) improved their resistance to RSV infection [10]. The treatment with NV1505 or PG1505 differentially regulated the respiratory innate antiviral immune response triggered by the activation of Toll-like receptor 3 (TLR3), improving respiratory interferon (IFN)-β, IFN-γ and interleukin (IL)-10 and reducing RSV replication and lung tissue damage. Furthermore, we were the first in demonstrating that nasal treatments with the postimmunobiotics NV1505 and PG1505 were also capable of reducing the susceptibility of infant mice to secondary pneumococcal colonization, bacteremia and inflammatory-mediated lung tissue injury produced after TLR3 activation or RSV infection [10]. Our recent findings indicated that NV1505 and PG1505 modulate the respiratory innate immune system by inducing the generation of activated/trained alveolar macrophages, which are capable of offering cross-protection against an unrelated pathogen (pneumococci) after a primary inflammatory (TLR3 activation) or infectious event (RSV) [18].

Due to the lack of studies evaluating the effect of postimmunobiotics on secondary pneumococcal pneumonia, in this work we set out to advance in the characterization of various aspects related to its potential application in humans, including: (a) the strain-specific properties in order to select the one(s) with the higher protective abilities; (b) the window of opportunity for postimmunobiotic intervention to achieve immunological protection; (c) the duration of the immunological priming; and (d) the ability of the treatments to protect against different pneumococcal serotypes.

## 2. Materials and Methods

### 2.1. Microorganisms

*Lacticaseibacillus rhamnosus* CRL1505, CRL498 and CRL576 were obtained from the CERELA-CONICET culture collection (Chacabuco 145, San Miguel de Tucumán, Argentina). The Laboratory of Bacterial Pathogenicity, part of the Faculty of Biological Sciences of Concepcion University (Concepcion, Chile), provided *L. rhamnosus* UCO25A. The Infection Biology Laboratory from Tucuman University and INSIBIO-CONICET (Tucuman, Argentina) provided *L. rhamnosus* IBL027. The strains UCO25A (human stomach) and IBL027 (human female genital tract) were selected because their immunomodulatory capacities. The strains CRL498 and CRL576 (human intestine) were selected as non-immunomodulatory controls. All lactobacilli cultures were kept freeze-dried. Lactobacilli were cultured for 12 h at 37 °C (final log phase) in de Man–Rogosa–Sharpe broth (MRS, Oxoid). The bacteria were harvested, washed three times with phosphate buffer saline (PBS, pH 7.2) and resuspended in sterile PBS.

Non-viable *L. rhamnosus* CRL1505 (NV1505), CRL498 (NV498), CRL576 (NV576), UCO25A (NVUCO25A) and IBL027 (NVIBL027) were obtained by tyndallization in a water bath at 80 °C for 30 min as described previously [10]. The lack of bacterial growth was confirmed using MRS agar plates. Peptidoglycans from *L. rhamnosus* CRL1505 (PG1505), CRL498 (PG498), CRL576 (PG576), UCO25A (PGUCO25A) and IBL027 (PGIBL027) were obtained as described previously [18,19]. Briefly, bacteria were grown in MRS broth for 18 h at 37 °C, washed 3 times with sterile PBS and lyophilized. Lactobacilli were resuspended in sterile water and lysed by sonication. The cell wall was processed, and pure peptidoglycans obtained for each individual strain were washed and lyophilized until use.

### 2.2. Animals and Treatments

Infant (3-week-old) BALB/c mice were obtained from the closed colony kept at CERELA-CONICET (San Miguel de Tucumán, Argentina). Animals were housed in plastic cages at room temperature and the assays for each parameter studied were performed on 5–6 mice per group for each time point. Non-viable *L. rhamnosus* strains were administered by the nasal route to infant mice for two consecutive days at a dose of 10^8^ cells/mouse/day in 50 μL of PBS [10,18]. The peptidoglycans obtained from the different *L. rhamnosus* strains were nasally administered to infant mice for two consecutive days at a dose of 8 μg/mL in 50 μL of PBS as described previously [10,18]. The treated groups and the PBS-treated control group were fed a conventional balanced diet ad libitum.

This study was carried out in strict accordance with the recommendations found in the Guide for the Care and Use of Laboratory Animals within the Guidelines for Animal Experimentation of CERELA-CONICET. The CERELA-CONICET Institutional Animal Care and Use Committee prospectively approved this research under the protocol BIOT-CRL-18. All efforts were made to minimize the number of animals and their suffering. No signs of discomfort or pain were observed before mice reached the endpoint. No deaths were observed before mice reached the endpoint.

### 2.3. Poly(I:C) Administration and Respiratory Infections

The administration of the TLR3 agonist poly(I:C) was performed on day 3, after two days of treatment with non-viable lactobacilli (NVs) or their peptidoglycans (PGs). Mice received 50 μL of PBS containing 250 μg poly(I:C) (equivalent to 10 mg/kg body weight), administered dropwise via the nares [10,20,21]. Control animals received 50 μL of PBS. Mice received three doses of poly(I:C) or PBS with a 24 h rest period between each administration.

Human RSV strain A2 was grown in Vero cells as described previously [10,20,21]. Briefly, Vero cells were grown and infected with RSV. After infection, Vero cells were scraped, sonicated and cell debris was removed by centrifugation. Virus supernatant was sucrose density gradient purified and stored in 30% sucrose at −80 °C. For in vivo infection, mice were challenged with 10^6^ PFU of RSV by the nasal route [10,18]. Viral challenge was performed on day 3, after the two days treatments with NVs or PGs. Lung RSV titers and tissue damage were evaluated 3 days after viral infection. The RSV immunoplaque assay was performed as described previously [10,20,22]. Primary RSV anti-F (clones 131-2A; Chemicon, Osaki, Japan), anti-G (Mouse monoclonal [8C5 (9B6)] to RSV glycoprotein, Abcam, Cambridge, UK) and secondary horseradish peroxidase anti-mouse immunoglobulin (Anti-mouse IgG, HRP-linked Antibody #7076, Cell signaling Technology, Danvers, MA, USA) antibodies were used. Individual plaques were developed using a DAB substrate kit (ab64238, Abcam) following the manufacturer’s specifications. Results were expressed as log_10_ PFU/g of lung.

*S. pneumoniae* serotype 6B was grown, harvested, washed with PBS and its cell density adjusted to 4 × 10^7^ CFU/mL [10,18]. Challenge with pneumococci was performed five days after the last administration of poly(I:C) or RSV infection. Mice were sacrificed two days after *S. pneumoniae* infection. Lungs were excised, weighed and homogenized in sterile peptone water. Homogenates were diluted appropriately, plated in duplicate on blood agar and incubated for 18 h at 37 °C. *S. pneumoniae* was identified by standard techniques and the results were expressed as the log of CFU/g of lung or CFU/mL of blood.

### 2.4. Alternative Treatments and Infections

For evaluation of the different timing of *S. pneumoniae* infection, mice were treated for two consecutive days with NV1505 or PG1505, stimulated with poly(I:C) on day 3 for three consecutive days and finally challenged with pneumococci on days 1, 15, 30 or 40 after the last administration of poly(I:C). Mice were sacrificed two days after *S. pneumoniae* infection.

For evaluation of the ability of immunobiotic interventions to protect against different *S. pneumoniae* serotypes, mice were treated for two consecutive days with NV1505 or PG1505, then stimulated with poly(I:C) for three consecutive days and finally challenged with pneumococci serotypes 3, 6B, 14 and 19F five days after the last administration of poly(I:C). Mice were sacrificed two days after *S. pneumoniae* infection.

For evaluation of the window of opportunity for immunobiotic intervention to improve the protection against secondary pneumococcal pneumonia, NV1505 or PG1505 were administered to mice 4 days before, concomitantly or 2 days after the administration of poly(I:C). These three groups were compared with mice treated with NV1505 or PG1505 2 days before poly(I:C) challenge, which is the protocol used in the previous experiments (Section 2.3.). Mice were sacrificed two days after *S. pneumoniae* infection.

In all the experiments, the lung and blood pneumococcal counts were determined as described in the Section 2.3. The lung tissue damage and respiratory cytokines were measured as described below.

### 2.5. Lung Injury Parameters and Cytokine Concentrations

Bronchoalveolar lavage (BAL) samples were obtained as described previously [10]. Briefly, the trachea was exposed and intubated with a catheter, and 2 sequential lavages were performed in each mouse by injecting sterile PBS. The recovered fluid was centrifuged and frozen at −70 °C for subsequent analyses. Albumin content, a measure to quantitate increased permeability of the bronchoalveolar–capillary barrier, and lactate dehydrogenase (LDH) activity, an indicator of general cytotoxicity, were determined in the acellular BAL fluid as described previously [10,18].

Interferon (IFN)-β (Mouse IFN-beta ELISA Kit, sensitivity: 15.5 pg/mL), IFN-γ (Mouse IFN-gamma Quantikine ELISA Kit, sensitivity: 2 pg/mL) and IL-10 (Mouse IL-10 Quantikine ELISA Kit, sensitivity: 5.2 pg/mL) concentrations in BAL samples were measured with commercially available enzyme-linked immunosorbent assay (elisa) technique kits following the manufacturer’s recommendations (R&D Systems, Minneapolis, MN, USA).

### 2.6. Alveolar Macrophage Primary Cultures and Quantitative PCR Expression Analysis

Primary cultures of murine alveolar macrophages were performed as described previously [10,18]. Briefly, macrophages were obtained from infant mice via bronchoalveolar lavages and transferred to new sterile tubes, washed twice in sterile PBS and resuspended in supplemented RPMI 1640 medium. BAL cells were seeded in 24-well plates and incubated to promote adherence. Non-adherent cells were washed, and macrophages were maintained for 24 h before stimulation. Alveolar macrophages were stimulated with *S. pneumoniae* (multiplicity of infection [MOI] of 3) or RSV (MOI of 5). The mRNA was extracted from alveolar macrophages 12 h after RSV challenge for the evaluation of cytokines and antiviral factor gene expressions.

Two-step real-time quantitative PCR was performed to characterize the expression of *IFN-α, IFN-β, IFN-γ, Mx1, RNAseL, OAS1, IL-1α, IL-1β, TNF-α, IL-6, IL-10* and *IL-27* genes in cultured alveolar macrophages. Total RNA was isolated from each sample using TRIzol reagent (Invitrogen, Waltham, MA, USA). All cDNAs were synthesized using a Quantitect reverse transcription (RT) kit (Qiagen, Tokyo, Japan) according to the manufacturer’s recommendations. Real-time quantitative PCR was carried out using a 7300 real-time PCR system (Applied Biosystems, Warrington, UK) and the Platinum SYBR green qPCR SuperMix uracil-DNA glycosylase (UDG) with 6-carboxyl-X-rhodamine (ROX) (Invitrogen). The primers used in this work were described previously [18,23]. The PCR cycling conditions were 2 min at 50 °C followed by 2 min at 95 °C, and then 40 cycles of 15 s at 95 °C, 30 s at 60 °C and 30 s at 72 °C. The reaction mixtures contained 5 μL of sample cDNA and 15 μL of master mix, which included the sense and antisense primers. Expression of β-actin was used to normalize cDNA levels for differences in total cDNA levels in the samples.

### 2.7. Statistical Analysis

Experiments were performed in triplicate and results were expressed as mean ± standard deviation (SD). After verification of the normal distribution of data, 2-way ANOVA was used. Tukey’s test (for pairwise comparisons of the means) was used to test for differences between the groups. Differences were considered significant at *p* < 0.05.

## 3. Results

### 3.1. Effect of Non-Viable Lactobacilli Strains and Their Peptidoglycans against Secondary Pneumococcal Pneumonia after Poly(I:C) Treatment

**Figure 1:**
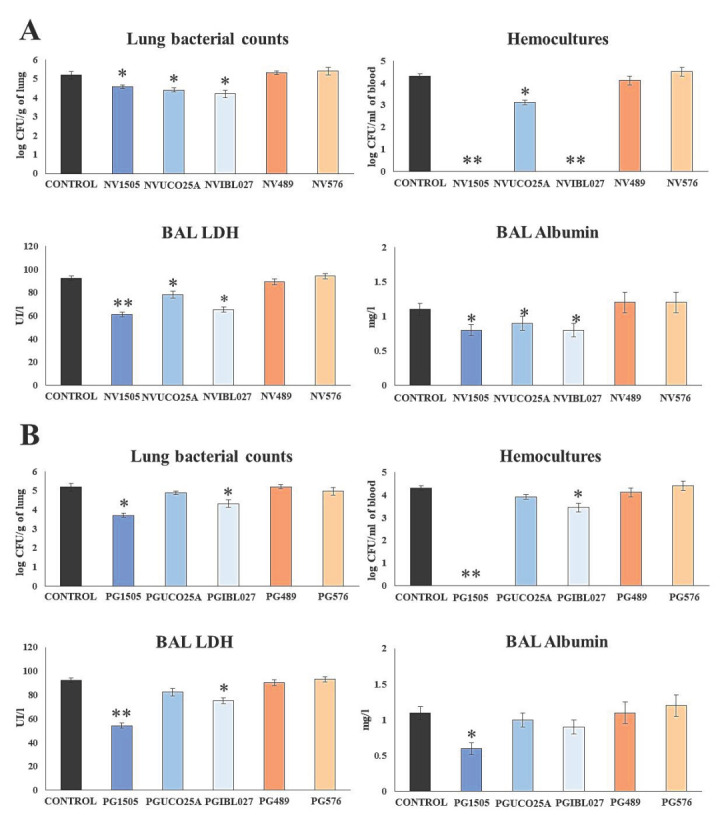
(**A**) Ability of nonviable (NV) *L. rhamnosus* CRL1505, CRL498, CRL576, UCO25A and IBL027 and (**B**) their peptidoglycans (PGs) to improve the resistance to secondary pneumococcal pneumonia after poly(I:C) treatment. Infant mice were nasally primed with NVs or PGs during two consecutive days, then stimulated with three once-daily doses of poly(I:C) and finally challenged with *S. pneumoniae* serotype 6B five days after the last administration of poly(I:C). Non-treated infant mice stimulated with poly(I:C) and then challenged with *S. pneumoniae* were used as controls. Lung bacterial cells counts, hemocultures, lactate dehydrogenase (LDH) activity and albumin concentrations in bronchoalveolar lavages (BAL) were determined on day 2 post-pneumococcal challenge. Asterisks indicate significant differences between treated and control groups; * (*p* < 0.05) and ** (*p* < 0.01).

Studies focused on the comparative evaluation of the immunomodulatory capacity of lactobacilli of the same species have shown that their impact on the immune system is a strain-specific characteristic [24,25,26,27]. Thus, we first aimed to evaluate whether the immunomodulatory properties of NV1505 and PG1505 in our respiratory superinfection models are strain-specific. For this purpose, comparative studies were carried out with different non-viable *L. rhamnosus* strains and their respective peptidoglycans. The immunomodulatory strains *L. rhamnosus* IBL027 [28,29] and UCO-25A [23,30] ,as well as non-immunomodulatory *L. rhamnosus* CRL489 and CRL576 [24], were used for the experiments. Infant mice were nasally treated with non-viable lactobacilli (NVs) or their peptidoglycans (PGs), stimulated with poly(I:C) and then challenged with *S. pneumoniae* as described in Section 2 (materials and methods). Two days after the pneumococcal challenge, lung and blood bacterial cell counts, as well as biochemical markers of lung injury, were evaluated (Figure 1).

The analysis of the pneumococcal counts in the lung and blood cultures evidenced differences between the evaluated treatments. As demonstrated previously [10], the NV1505 treatment reduced the bacterial counts in the lung and prevented the spread of *S. pneumoniae* into the blood (Figure 1A). Similarly, the NVUCO25A and NVIBL027 treatments significantly reduced *S. pneumoniae* counts in the lungs of infected infant mice. The NVIBL027 treatment prevented the dissemination of the respiratory pathogen to blood, while NVUCO25A induced a decrease in pneumococcal blood counts. On the other hand, the NV489 and NV576 treatments did not induce significant changes in the resistance to secondary pneumococcal infection when compared to the control group (Figure 1A).

The analysis of the biochemical parameters evaluating lung tissue damage showed that NV1505, NVUCO25A and NVIBL027 were effective in protecting infected mice against the cellular damage and alveolar–capillary barrier alterations, since the levels of BAL LHD activity and albumin concentration were significantly lower compared to the control group. The three treatments were equally efficient at reducing BAL albumin values, while NV1505 was more effective than NVUCO25A and NVIBL027 at decreasing BAL LHD activity (Figure 1 A). The NV489 and NV576 treatments did not induce significant changes in the biochemical parameters of lung damage when compared with the control group (Figure 1A).

The effect of peptidoglycans was studied in a similar way, as shown in Figure 1B. When the pneumococcal counts were evaluated in the lung and blood cultures, only the PG1505 and PGIBL027 treatments were able to significantly decrease both parameters. However, PG1505 was more efficient than PGIBL027 at protecting against pneumococcal dissemination. The other treatments did not induce significant differences with respect to the control group. The study of BAL albumin concentration showed that only the PG1505 treatment was able to significantly decrease this parameter when compared to controls. It was also observed that infant mice treated with PG1505 or PGIBL027 had significantly lower BAL LDH activity values compared to the control group (Figure 1B). However, PG1505 was more efficient than PGIBL027 at reducing LDH activity in BAL. The PGUCO25A, PG489 and PG576 treatments were not able to induce modifications of the biochemical parameters used for the evaluation of lung tissue damage (Figure 1B).

**Figure 2:**
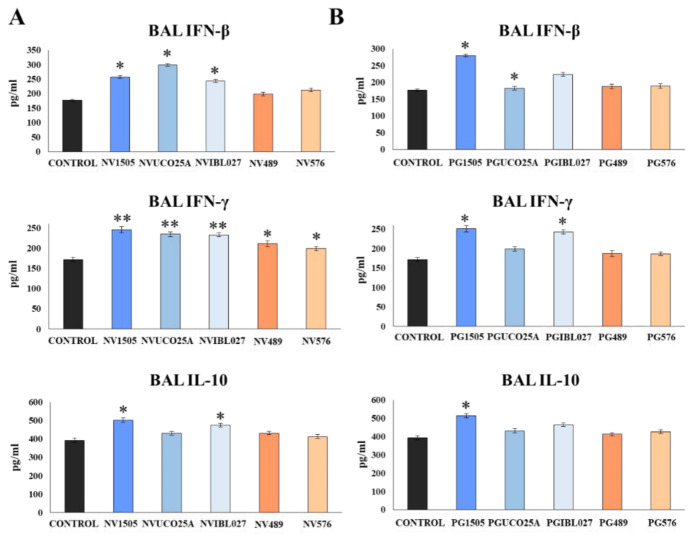
(**A**) Role of interferon (IFN)-β, IFN-γ and interleukin (IL)-10 in the ability of non-viable (NVs) *L. rhamnosus* CRL1505, CRL498, CRL576, UCO25A and IBL027 and (**B**) their peptidoglycans (PGs) to improve resistance to secondary pneumococcal pneumonia after poly(I:C) treatment. Infant mice were nasally primed with NVs or PGs for two consecutive days, then stimulated with three once-daily doses of poly(I:C) and finally challenged with *S. pneumoniae* serotype 6B five days after the last administration of poly(I:C). Non-treated infant mice stimulated with poly(I:C) and then challenged with *S. pneumoniae* were used as controls. The levels of IFN-β, IFN-γ and IL-10 in bronchoalveolar lavages (BAL) were determined on day 2 post-pneumococcal challenge. Asterisks indicate significant differences between treated and control groups; * (*p* < 0.05) and ** (*p* < 0.01).

Our previous results indicated that IFN-β, IFN-γ and IL-10 are involved in the beneficial immunoregulatory effects of nasally administered NV1505 and PG1505 in the context of respiratory superinfection [10,18]. Here, we evaluated the influence of the different treatments on the concentration of these three cytokines in BAL samples two days after the infection with *S. pneumoniae*, as shown in Figure 2A.

NV1505, NVUCO25A and NVIBL027 treatments were equally effective in increasing BAL IFN-β levels compared to the control group, while NV489 and NV576 did not induce significant changes in the concentrations of this cytokine. The five treatments increased BAL IFN-γ levels with respect to the control group. However, NV1505, NVUCO25A and NVIBL027 were more efficient than NV489 and NV576 to increase the concentration of IFN-γ in the respiratory tract during the secondary pneumococcal infection. On the other hand, only NV1505 and NVIBL027 increased the levels of BAL IL-10, while the remaining treatments did not induce significant differences with respect to the control group (Figure 2A). The concentrations of BAL IFN-β, IFN-γ and IL-10 were also evaluated for PGs treatments (Figure 2B). PG1505 and PGIBL027 were equally effective in increasing the levels of BAL IFN-β and IFN-γ compared to the control group, but only the PG1505 treatment increased BAL IL-10 levels. Of note, PGUCO25A, PG489 and PG576 did not induce significant modifications in the concentrations of BAL IFNs or IL-10 when compared to controls (Figure 2B).

### 3.2. Effect of Non-Viable Lactobacilli Strains and Their Peptidoglycans against Secondary Pneumococcal Pneumonia after RSV Infection

**Figure 3:**
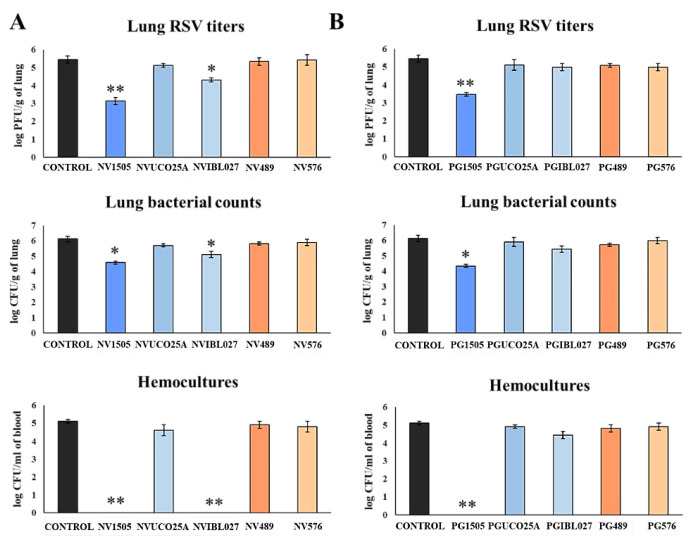
(**A**) Ability of non-viable (NV) *L. rhamnosus* CRL1505, CRL498, CRL576, UCO25A and IBL027 and (**B**) their peptidoglycans (PGs) to improve the resistance to secondary pneumococcal pneumonia after primary infection with respiratory syncytial virus (RSV). Infant mice were nasally primed with NVs or PGs for two consecutive days, then challenged with RSV on day 3 and finally infected with S. *pneumoniae* serotype 6B five days after the viral infection. Non-treated infant mice infected with RSV and challenged with *S. pneumoniae* were used as controls. Lung RSV titers were evaluated on day 2 post-viral challenge while lung bacterial cells counts and hemocultures were determined on day 2 post-pneumococcal challenge. Asterisks indicate significant differences between treated and control groups; * (*p* < 0.05) and ** (*p* < 0.01).

We also aimed to demonstrate the differences between the NV and PG treatments in their capacities to protect against the secondary pneumococcal pneumonia produced in infant mice after infection with RSV. For this purpose, mice were nasally primed with NVs or PGs, infected with RSV and, five days after the viral infection, challenged with *S. pneumoniae*. Lung RSV titers were evaluated on day 2 post-viral challenge while pneumococcal colonization, bacteremia and BAL markers of injury were evaluated on day 2 post-*S. pneumoniae* challenge (Figure 3).

In all the experimental groups, RSV was detected in the lungs of infected mice (Figure 3). As we described previously [10], NV1505 and PG1505 were able to significantly reduce RSV titers in lungs of infected mice. Of note, among the other NVs treatments, only NVIBL027 reduced lung viral titers when compared to controls (Figure 3A). In addition, both NV1505 and PG1505 significantly reduced pneumococcal counts in lungs and prevented dissemination of the respiratory pathogen into the blood (Figure 3), which was in line with the results obtained in the poly(I:C) model (Figure 1). The NVIBL027 treatment was as efficient as the NV1505 treatment at reducing pneumococcal colonization and avoiding dissemination, but the PGIBL027 treatment did not induce significant differences with respect to the control group (Figure 3B). Treatment with the other NVs and PGs did not exert any protection against RVS primary infection or secondary pneumococcal pneumonia (Figure 3).

### 3.3. Effect of Non-Viable Lactobacilli Strains and Their Peptidoglycans on Alveolar Macrophage Cytokine Profiles in Response to Infectious Challenges

**Figure 4:**
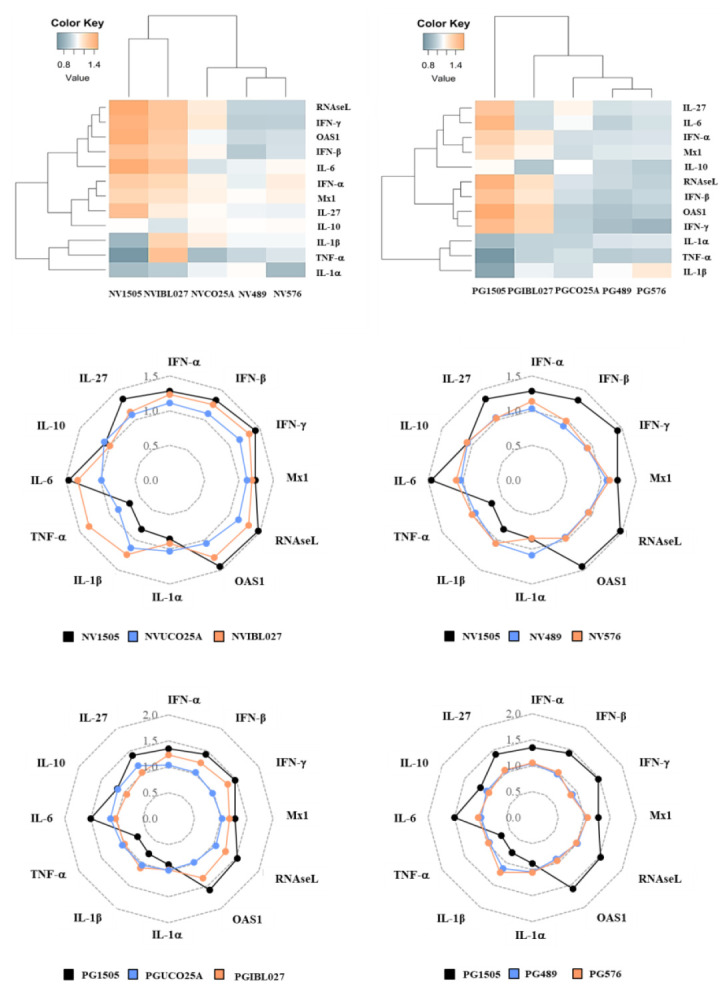
Effect of non-viable (NV) *L. rhamnosus* CRL1505, CRL498, CRL576, UCO25A and IBL027 and their peptidoglycans (PGs) on alveolar macrophage cytokines and antiviral factor profiles in response to respiratory syncytial virus (RSV) challenge. Infant mice were nasally primed with NVs or PGs for two consecutive days. Alveolar macrophages were isolated from infant mice on the third day and challenged in vitro with RSV. The expression of *IFN-α*, *IFN-β*, *IFN-γ*, *Mx1*, *RNAseL*, *OAS1*, *IL-1α*, *IL-1β*, *TNF-1α*, *IL-6*, *IL-10* and *IL-27* genes were evaluated in alveolar macrophages by quantitative PCR (qPCR) after 12 h. The results represent data from three independent experiments. The multiple comparisons of the magnitude of the fold expression changes with respect to the control are shown for the NV1505 vs. NVUCO25A vs. NVIBL027, NV1505 vs. NV489 vs. NV576, PG1505 vs. PGUCO25A vs. PGIBL027 and PG1505 vs. PG489 vs. PG576 groups.

Taking into consideration the relevant role played by the cytokines produced by alveolar macrophages in the beneficial effects of immunobiotics demonstrated recently by our group [18], we next aimed to evaluate the impact of the different NV and PG treatments on the cytokines produced by alveolar macrophages in response to RSV and *S. pneumoniae* challenges. In the first set of experiments, mice were nasally primed with the different treatments for two consecutive days as described in materials and methods, and alveolar macrophages were isolated on the third day. Primary cultures of alveolar macrophages were challenged in vitro with RSV (Figure 4) and the expression of several immunological factors were evaluated after 12 h. As shown in Figure 4, the treatment with NV1505 significantly increased the expression of *IL-6*, *IFN-α*, *IFN-β*, *IFN-γ*, *RNAseL*, *OAS1* and *IL-27* in alveolar macrophages challenged with RSV. In addition, NV1505 induced a repression of *TNF-α*, *IL-1α* and *IL-1β*. Similar results were obtained for the NVIBL027 treatment when the expression levels of *IL-6*, *IFN-α*, *IFN-β*, *RNAseL*, *OAS1* and *IL-27* were evaluated. However, the inflammatory cytokines *TNF-α* and *IL-1β* were significantly increased in alveolar macrophages from the NVIBL027 group when compared to controls (Figure 4). The NVUCO25A, NV489 and NV576 treatments did not induce changes in cytokine mRNAs levels after RSV challenge when compared to controls, although alveolar macrophages from NVUCO25A had a tendency to express more IFNs and antiviral factors than control macrophages. These differences were visualized clearly using a heat-map analysis in which the groups were separated based on the magnitude of the differences of all factors studied with respect to control. This analysis showed that NV1505 and NVIBL027 clustered separately from NVUCO25A and the non-immunomodulatory treatments NV489 and NV576 (Figure 4).

When the cytokine profile of alveolar macrophages challenged with RSV obtained from mice treated with PG1505 was analyzed, similar results to the NV1505 treatment were observed (Figure 4). Interestingly, alveolar macrophages from PGIBL027-treated mice had enhanced expressions of *IFN-α*, *IFN-β*, *IFN-γ*, *RNAseL*, *Mx1* and *OAS1,* but no modifications in *TNF-α*, *IL-6*, *IL-1α*, *IL-1β* or the immunoregulatory cytokines were observed when compared to controls. In addition, cytokine profiles in alveolar macrophages from PGUCO25A, PG489 and PG576 groups were similar to the controls (Figure 4). The heat-map analysis clearly showed that PG1505 has unique immunomodulatory properties in alveolar macrophages that are not shared by the peptidoglycans obtained from immunomodulatory *L. rhamnosus* strains (Figure 4).

**Figure 5:**
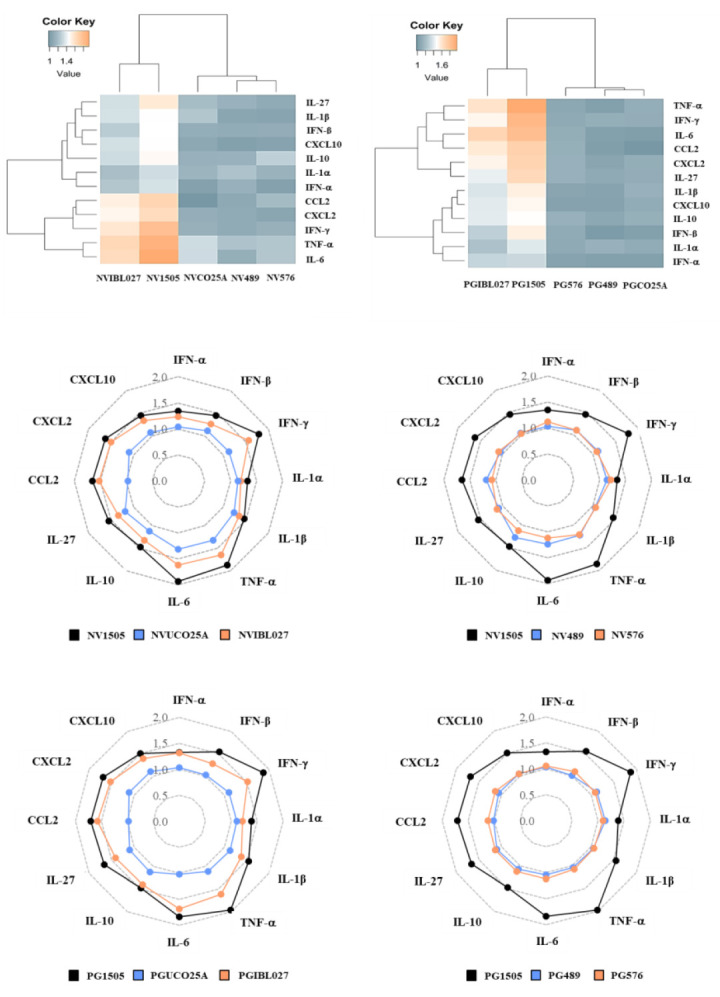
Effect of non-viable (NV) *L. rhamnosus* CRL1505, CRL498, CRL576, UCO25A and IBL027 and their peptidoglycans (PGs) on alveolar macrophage cytokine profiles in response to secondary *S. pneumoniae* infection. Infant mice were nasally primed with NVs or PGs for two consecutive days and challenged with the respiratory syncytial virus (RSV). Alveolar macrophages isolated from infant mice were stimulated in vitro with *S. pneumoniae*. The production of *IFN-β*, *IFN-γ*, *IL-6*, *IL-12*, *IL-10*, *IL-27*, *TNF-α*, *IL-1β*, *CCL2*, *CCL3*, *CXCL2* and *CXCL10* was evaluated in alveolar macrophage culture supernatants after 24 h. The results represent data from three independent experiments. The multiple comparisons of the magnitude of the fold expression changes with respect to the control are shown for the NV1505 vs. NVUCO25A vs. NVIBL027, NV1505 vs. NV489 vs. NV576, PG1505 vs. PGUCO25A vs. PGIBL027 and PG1505 vs. PG489 vs. PG576 groups.

In the second set of experiments, mice were nasally primed with the different NV and PG treatments for two consecutive days and then challenged with RSV. Five days after the viral challenge, alveolar macrophages were isolated. Primary cultures of alveolar macrophages were challenged in vitro with *S. pneumoniae* (Figure 5) and the expression of several immunological factors were evaluated after 12 h.

It was observed that in alveolar macrophages obtained from NV1505-treated mice the expression of all the factors analyzed (*IFN-α*, *IFN-β*, *IFN-γ*, *TNF-α*, *IL-6*, *IL-1α*, *IL-1β*, *CCL2*, *CXCL2*, *CXCL10*, *IL-10* and *IL-27*) were significantly higher than controls. The NVIBL027 treatment increased all the cytokine mRNAs evaluated in proportions similar to those observed for NV1505, while NVUCO25A increased only the expressions of *TNF-α*, *IL-6*, *IL-1α* and *IL-1β* (Figure 5). Interestingly, it was observed that both PG1505 and PGIBL027 exerted similar modifications in cytokine profiles when compared to NV1505 and NVIBL027, respectively (Figure 5). In contrast, alveolar macrophages obtained from PGUCO25A-treated mice had no differences with controls when the inflammatory and regulatory cytokines expressions were compared. The treatments with NV489, NV576 or their peptidoglycans did not exert any significant change in alveolar macrophage cytokine profiles in response to bacterial infection when compared to controls (Figure 5). These differences were visualized in heat-map analysis; PG1505 and PGIBL027 clustered separately from the other treatments (Figure 5).

### 3.4. NV1505 and PG1505 Protect Infant Mice from Secondary Pneumococcal Pneumonia up to 30 Days after the Primary Viral Inflammation

**Figure 6:**
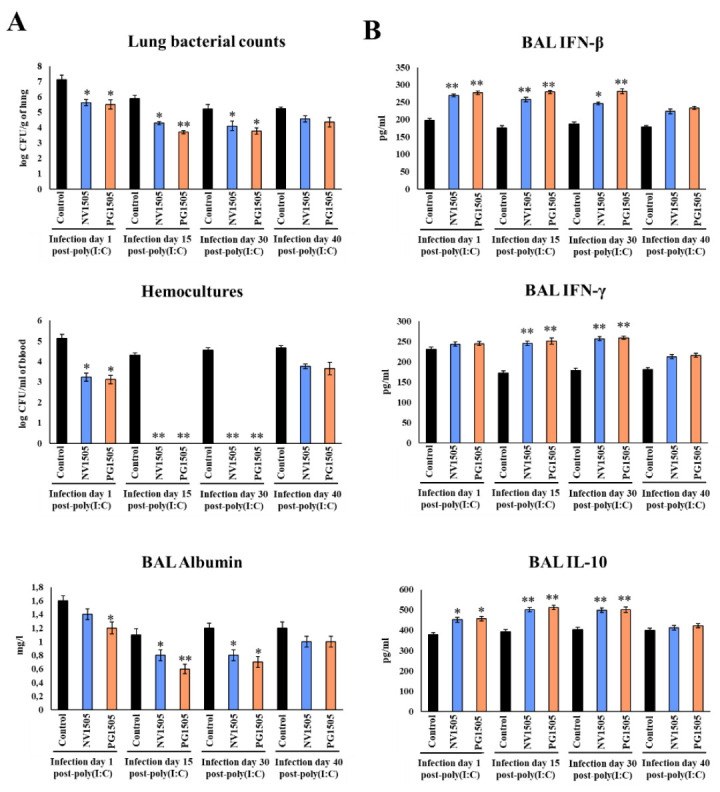
Ability of non-viable *L. rhamnosus* CRL1505 (NV) and its peptidoglycan (PG) to enhance resistance to secondary pneumococcal pneumonia after poly(I:C) treatment over time. Infant mice were nasally primed with NV1505 or PG1505 for two consecutive days. They were stimulated with three once-daily doses of poly(I:C) and finally challenged with *S. pneumoniae* on days 1, 15, 30 and 40 after the last poly(I:C) administration. (**A**) Lung bacterial cells counts, hemocultures and albumin concentrations in bronchoalveolar lavages (BAL) as well as (**B**) the levels of interferon (IFN)-β, IFN-γ and interleukin (IL)-10 in BAL were determined on day 2 post-pneumococcal challenge. The results represent data from three independent experiments. Asterisks indicate significant differences between treated and control groups; * (*p* < 0.05) and ** (*p* < 0.01).

We aimed to evaluate the duration of protection against secondary pneumococcal pneumonia after immunological priming with NV1505 or PG1505. For this purpose, infant mice were treated nasally with NV1505 or PG1505, stimulated with poly(I:C) and challenged with *S. pneumoniae* on days 1, 15, 30 and 40 after the last poly(I:C) administration. Lung and blood bacterial cell counts, biochemical markers of lung injury and respiratory cytokine levels were evaluated 2 days after each pneumococcal challenge (Figure 6).

Similar to the results observed in mice challenged with *S. pneumoniae* on day 5 after the last poly(I:C) administration (Figure 1), NV1505 and PG1505 were able to significantly reduce the pneumococcal cells counts in lungs, prevent its dissemination into blood and reduce BAL albumin levels in mice challenged with pneumococci on days 15 and 30 after poly(I:C) stimulation (Figure 6A). Those beneficial effects were associated with significantly enhanced levels of BAL IFN-β, IFN-γ and IL-10 (Figure 6B), which reached values that were not different from that observed in NV1505 and PG1505 groups infected with *S. pneumoniae* on day 5 after the last poly(I:C) administration (Figure 1). In contrast, all the parameters evaluated in mice treated with NV1505 or PG1505 were not different from controls when the pneumococcal challenge was produced on day 40 after the last poly(I:C) administration (Figure 6). Interestingly, although both NV1505 and PG1505 treatments were able to reduce lung and blood pneumococcal counts, diminish BAL albumin levels and increase BAL IFN-β and IL-10 when the pneumococcal challenge was produced on day 1 after the last poly(I:C) administration, the values of those parameters were not improved as observed in the groups challenged on days 5 (Figure 1), 15 or 30 (Figure 6). Furthermore, no modifications in BAL IFN-γ concentrations were observed in NV1505 or PG1505 groups when compared to controls (Figure 6).

We also evaluated the ability of the different NV and PG treatments to confer protection against secondary pneumococcal pneumonia after 30 days (Supplementary Figure S1). Only the NVIBL027 treatment was able to reduce pneumococcal counts in lung and blood, diminish BAL albumin concentrations and increase BAL IFN-β, IFN-γ and IL-10 levels as done by NV1505 and PG1505.

### 3.5. The Window of Opportunity for NV1505 and PG1505 Intervention to Achieve Immunological Protection against Secondary Pneumococcal Pneumonia

**Figure 7:**
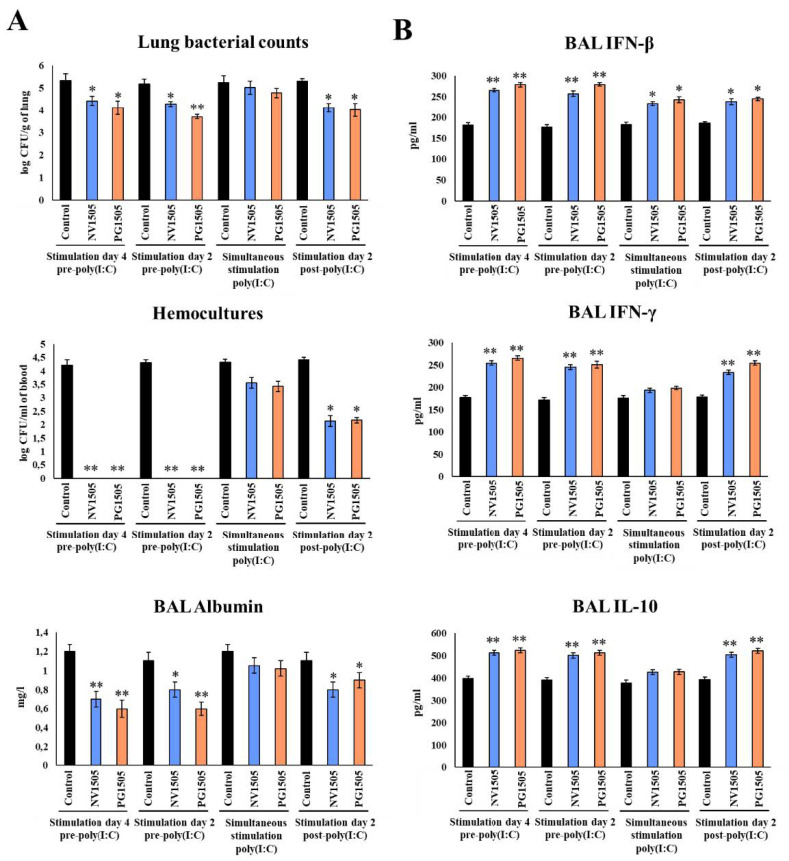
Window of opportunity to improve the protection against secondary pneumococcal pneumonia with NV1505 or PG1505 treatments. Infant mice were nasally treated with NV1505 or PG1505 2 and 4 days before, concomitantly or 2 days after the administration of poly(I:C). In all groups, *S. pneumoniae* infection was induced on day 5 after the last poly(I:C) administration. (**A**) Lung bacterial cell counts, hemocultures and albumin concentrations in bronchoalveolar lavages (BAL) as well as (**B**) the levels of interferon (IFN)-β, IFN-γ and interleukin (IL)-10 BAL were determined on day 2 post-pneumococcal challenge. The results represent data from three independent experiments. Asterisks indicate significant differences between treated and control groups; * (*p* < 0.05) and ** (*p* < 0.01).

We next aimed to study the window of opportunity for improving the protection against secondary pneumococcal pneumonia with NV1505 or PG1505 treatments. Thus, NV1505 and PG1505 were administered to mice 4 days before, concomitantly or 2 days after the administration of poly(I:C). These three groups were compared with mice treated with NV1505 or PG1505 2 days before poly(I:C) challenge, which is the protocol used in the previous experiments. In all the groups, *S. pneumoniae* infection was induced on days 5 after the last poly(I:C) administration (Figure 7). Both the administration of NV1505 and PG1505, 4 or 2 days before poly(I:C) stimulation, were equally effective in reducing lung and blood pneumococcal counts and BAL albumin levels (Figure 7A) as well as increasing BAL IFN-β, IFN-γ and IL-10 (Figure 7B) after challenge with *S. pneumoniae*. In contrast, when NV1505 or PG1505 were administered concomitantly with poly(I:C), the pathogen colonization and dissemination, the marker of lung in injury and the concentrations of BAL cytokines were not different from control mice (Figure 7).

When the NV1505 and PG1505 treatments were administered 2 days after the challenge of poly(I:C), the lung pneumococcal counts and BAL albumin concentrations (Figure 7A), as well as the BAL IFN-γ and IL-10 levels (Figure 7B), were modulated with a similar magnitude as that observed in mice treated with NV1505 and PG1505 4 or 2 days before poly(I:C) stimulation. Of note, although the blood pneumococcal cell counts were reduced and the BAL IFN-β levels were increased, the values of those parameters did not reach the levels found in the groups of mice treated with NV1505 and PG1505 4 or 2 days before poly(I:C) stimulation (Figure 7).

We performed experiments with NVs and PGs administered 2 days after poly(I:C) stimulation in order to characterize the effect of the distinct *L. rhamnosus* strains (Supplementary Figure S2). No significant differences were observed when most of the treatments were compared to controls. Of note, the NVIBL027 treatment was not as effective as the NV1505 and PG1505 treatments at improving protection against secondary pneumococcal infection when administered 2 days after poly(I:C) challenge. Furthermore, the values of BAL albumin in mice treated with NVIBL027 had a tendency to be higher than those observed in control mice.

### 3.6. NV1505 and PG1505 Protect Infant Mice from Secondary Pneumococcal Pneumonia Produced by Different S. pneumoniae Serotypes

**Figure 8:**
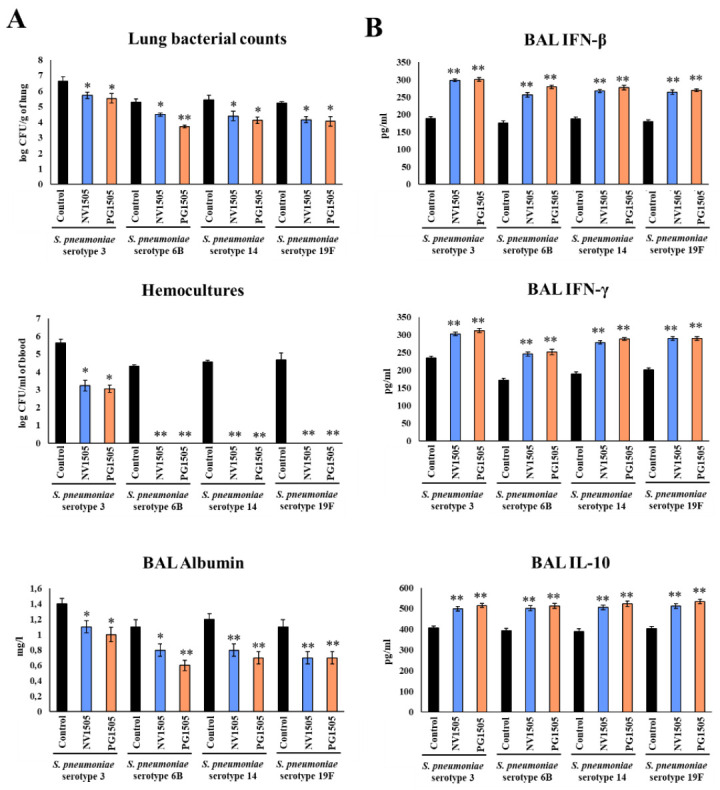
Ability of non-viable *L. rhamnosus* CRL1505 (NV) and its peptidoglycan (PG) to enhance resistance to secondary pneumococcal pneumonia produced by *S. pneumoniae* serotypes after poly(I:C) stimulation. Infant mice were nasally primed with NV1505 or PG1505 for two consecutive days, then stimulated with three once-daily doses of poly(I:C) and finally challenged with *S. pneumoniae* serotype 3, 6B, 14 or 19F five days after the last administration of poly(I:C). Non-treated infant mice stimulated with poly(I:C) and then challenged with the respective strains of *S. pneumoniae* were used as controls. (**A**) Lung bacterial cell counts, hemocultures and albumin concentrations in bronchoalveolar lavages (BAL) as well as (**B**) the levels of interferon (IFN)-β, IFN-γ and interleukin (IL)-10 in BAL were determined on day 2 post-pneumococcal challenge. The results represent data from three independent experiments. Asterisks indicate significant differences between treated and control groups; * (*p* < 0.05) and ** (*p* < 0.01).

Finally, we investigated the capacity of NV1505 and PG1505 to protect against secondary pneumococcal pneumonia produced by different *S. pneumoniae* serotypes. For this purpose, infant mice were nasally treated with NVs or PGs, stimulated with poly(I:C) and then challenged with *S. pneumoniae* serotypes 3, 6B, 14 or 19F. Two days after pneumococcal challenge, lung and blood bacterial cell count, biochemical markers of lung injury and respiratory cytokines were evaluated (Figure 8). In our tests, the administration of both NV1505 and PG1505 were effective in reducing *S. pneumoniae* counts in lung and blood, diminishing BAL albumin and improving BAL IFN-β, IFN-γ and IL-10 for serotypes 14 and 19F (Figure 8), doing so as efficiently as observed with serotype 6B (Figure 1) [10]. Interestingly, the NV1505 and PG1505 treatments were as effective in increasing the levels of BAL IFN-β, IFN-γ and IL-10 in mice infected with *S. pneumoniae* serotype 3 as observed in the other pneumococcal serotypes (Figure 8B). However, although these treatments reduced the pathogen counts in lung and blood as well as BAL albumin in comparison to controls, the values of the three parameters were higher than those observed in NV1505- and PG1505-treated mice infected with serotypes 6B, 14 or 19F (Figure 8).

We selected *S. pneumoniae* serotype 3 to assess the effect of the different NVs and PGs (Supplementary Figure S3). No significant differences were found between the NV and PG treatments in comparison with controls, with the exception of NVIBL027 which reduced pneumococcal counts, decreased BAL albumin concentrations and improved the levels of BAL IFN-β, IFN-γ and IL-10.

## 4. Discussion

Research on probiotic and immunobiotic bacteria over the past decades have consistently demonstrated that the beneficial effects of such microorganisms are strain-specific characteristics [24,25,26,27]. Our own studies evaluating the impact of immunobiotics on the intestinal immune response triggered by the activation of TLR3 revealed the different capacity of strains of the species *L. rhamnosus*, *L. plantarum* [24,25,26,27] and *Ligilactobacillus salivarius* [24,25,26,27] to beneficially modulate the innate antiviral immune response. We have also demonstrated that nasally administered peptidoglycans derived from lactobacilli strains are not equally effective in improving protection against *S. pneumoniae* respiratory challenge in adult immunocompromised malnourished mice [24,25,26,27]. Furthermore, our recent comparative study evaluating the effect of nasally administered heat-killed *L. rhamnosus* CRL1505 and CRL489 and their purified peptidoglycans revealed significant differences in their capacities to modulate respiratory immune responses in the context of RSV–pneumococcal superinfection in infant mice [10,20,21]. As CRL489 is not an immunomodulatory strain, these results were expected. Thus, further comparative studies of NV1505 and PG1505 with other immunomodulatory and non-immunomodulatory *L. rhamnosus* strains were necessary to convincingly demonstrate that the immunobiotic effect is strain-dependent in the context of respiratory superinfections. We performed such demonstration in the present work by showing the different capacity of immunomodulatory (CRL1505, IBL027 and UCO25A) and non-immunomodulatory (CRL489 and CRL576) *L. rhamnosus* to regulate respiratory TLR3-mediated immunity and protect against primary RSV and secondary pneumococcal infections.

We demonstrated that nonviable immunomodulatory UCO25A was not as effective as NV1505 and NVIBL027 at regulating respiratory immunity. Moreover, among the five peptidoglycans evaluated, only PG1505 induced a beneficial immunoregulatory effect. Similar to NV1505 and PG1505, the NVIBL027 treatment was able to modulate alveolar macrophage functions to improve resistance against infections. We reported previously that NV1505 or PG1505 modulated the production of cytokines by alveolar macrophages in response to both *S. pneumoniae* and RSV challenges, and that the depletion of alveolar macrophages by clodronate liposomes at the time of NV1505 or PG1505 nasal priming abolished their ability to enhance protection against the respiratory superinfection [10,20,21]. Like NV1505 and PG1505, the nasal treatment of infant mice with NVIBL027 improved the production of IFN-β and IFN-γ in the respiratory tract, factors that are well-known to be involved in protection against RSV [31] and pneumococci [32,33]. The profile of cytokines induced by NVIBL027 in alveolar macrophages was similar to that observed for NV1505 and PG1505, with the exception of *TNF-α* and *IL-1β* which were significantly higher for NVIBL027. These results indicate that NVIBL027 would induce a more proinflammatory profile in alveolar macrophages, which is in line with our previous studies demonstrating the ability of *L. rhamnosus* IBL027 to function as a potent mucosal adjuvant. Bacterium-like particles derived from the IBL027 strain have been shown to improve antigen-specific humoral and cellular adaptive immune responses when administered by nasal [34] or oral [10,20,21] routes. Of note, although NVIBL027 was efficient in modulating respiratory innate immunity, this effect was not observed for its purified peptidoglycan PGIBL027. The reason for this phenomenon is still unclear, but similar results have been reported with other strains of lactobacilli. It was shown that the nasal treatment with heat-killed *L. plantarum* CRL1506 [10,20,21] or *Limosilactobacillus reuteri* F275 [15,16] significantly increased the protection of mice against *S. pneumoniae* or pneumonia virus of mice infections, respectively, while their purified peptidoglycans did not enhance protection.

In this work, we also advanced the characterization of aspects related to the potential application of NV1505 and PG1505 in humans, including the ability of the treatments to protect against different pneumococcal serotypes, the window of opportunity for immunobiotic intervention to achieve immunological protection and the duration of immunological priming.

The capsule of *S. pneumoniae* is critical for the induction of protective immunity. In fact, available vaccines against pneumococci are developed by considering the most prevalent capsule serotypes causing infections in humans. Thus, pneumococcal vaccines provide serotype-specific protection against the infection produced by this pathogen, including invasive pneumococcal disease [35,36,37]. As the infections produced by the pneumococcal serotypes included in the vaccine formulations decreased, previously rare serotypes became prevalent in the nasal carriage and in invasive disease, altering the epidemiology of pneumococcal infections, a phenomenon called “serotype replacement” [36,37]. A method for avoiding this disadvantage of pneumococcal vaccines is the constant replacement and inclusion of new serotypes in vaccine formulations, which necessarily enhances the cost and complexity of production, making vaccines less accessible [35,37]. Thus, great efforts are being made to develop serotype-independent protective vaccines against pneumococci. Another alternative is the improvement of innate immunity, which may provide natural protection against pneumococci in a serotype-specific manner.

Commensal and probiotic bacteria have been shown to be efficient in increasing protection against primary pneumococcal infections. It was shown that the commensal *Streptococcus mitis*, which is a microorganism that that exhibits immune cross-reactivity with *S. pneumoniae*, can enhance protection against primary lung pneumococcal infection in mice [38]. Interestingly, *S. mitis* modulated respiratory immunity and increased protection against the pneumococcal serotypes 2 and 4. We have also reported that the nasal administration of the respiratory commensal bacteria *Dolosigranulum pigrum* 040417 improved the resistance to *S. pneumoniae* lung infection produced by serotypes 6B, 14 and 3 in infant mice by modulating innate immunity [39]. In line with these previous works, we demonstrated here for the first time that the treatment of mice with NV1505 and PG1505 increased the protection against secondary pneumococcal infection produced by different serotypes. In our hands, the two postimmunobiotic treatments enhanced resistance to secondary pneumococcal pneumonia induced by serotypes 3, 6B, 14 and 19F. Of note, the two treatments were less efficient in improving resistance against serotype 3 than against the other serotypes. These results are probably related to the different degrees of virulence associated with *S. pneumoniae* serotypes in mice. It was postulated that the thickness of the pneumococcal capsule is related to virulence and inflammatory capacities [40,41]. Serotype 3 possessed a larger capsule than serotype 19F and, therefore, was more virulent for mice [39,40,41]. A study determined that mice challenged with serotypes 3 and 4 developed severe clinical manifestations associated with necrosis, edema and cellular infiltration in the lungs by day 3 post-infection, while animals infected with serotype 19F started to recover [40]. Comparative studies with pneumococcal serotypes 3 and 8 evaluating gene expression in the lungs of mice revealed that serotype-3-infected animals exhibited a more exuberant inflammatory response than mice challenged with serotype 8 [41]. Interestingly, serotype-3-infected CXCR3-deficient mice recruited more macrophages to their lungs and had a less exuberant lung inflammatory response than wild type mice, which is in line with the results presented here; this suggests a key role for macrophages in the more efficient control of pneumococci induced by NV1505 and PG1505. Our future studies will investigate the clearance kinetics of the different pneumococcal serotypes, as well as parameters of the innate and adaptive immune response, to further characterize the beneficial effect of postimmunobiotics.

When a host is exposed to a respiratory virus, its replication in the mucosa occurs primarily during the first week of infection. The initial viral-replication phase is usually followed by a cascade of inflammatory events that are intended to eliminate the virus but, if unregulated, contribute to pathogenesis [10,11,12,13,14]. The course of viral infection and inflammation offers clinicians separate windows of specific time intervals to administer either antiviral or immunomodulatory therapy. Thus, we also aimed to investigate the window of opportunity for a postimmunobiotic intervention to influence antiviral response and positively impact protection against secondary pneumococcal pneumonia. When NV1505 or PG1505 were administered concomitantly or after poly(I:C), no changes or reduced effect were observed in resistance to secondary *S. pneumoniae* infection compared to the administration of postimmunobiotics 2 days before TLR3 activation. This may be due to the fact that, in these circumstances, the immune system is overwhelmed by the activation of the TLR3 signaling pathway, thus being unable to sense postimmunobiotics. Of note, nasal priming with NV1505 or PG1505 could have potentiated respiratory inflammation, exerting an effect opposite to that expected. However, the treatments were not additional stressors for the immune system, since they did not induce differences or only slightly reduced the levels of the parameters evaluated with respect to the controls. In contrast, when NVIBL027 was administered after poly(I:C) stimulation, it lost its capacity to reduce pneumococcal counts or increase the levels of BAL cytokines. Moreover, in this circumstance, the NVIBL027-treated mice showed a tendency toward higher levels of BAL albumin. This could be related to the proinflammatory properties of NVIBL027 that function as an additional inflammatory stress for lungs. This fact also highlights the strain-specific properties of postimmunobiotics.

When the NV1505 and PG1505 treatments were administered 4 days before stimulation with poly(I:C), they were equally efficient in improving the response to secondary pneumococcal infection compared to administration 2 days before TLR3 activation. Our results showed that prophylactic administration is efficient in improving the response to respiratory superinfection with a margin of up to four days before TLR3 activation. This has important practical implications since, as it is a prophylactic administration, it is not possible to predict when the host will face the viral infection. Therefore, it is possible to speculate that nasal priming with NV1505 or PG1505 for healthy hosts during, e.g., winter seasons or the presence of an epidemic could help to improve their defences if they encounter the virus, and also decrease viral shedding in the community. These treatments would also help to reduce the severity of secondary bacterial pneumonia. Furthermore, we demonstrated here that NV1505 and PG1505 can enhance the response against *S. pneumoniae* secondary infection up to 30 days after the TLR3-mediated inflammation. We speculate that this effect is induced by the generation of alveolar macrophages with a trained immunity phenotype.

Research from the last several years has demonstrated that, after a primary challenge, macrophages can develop a nonspecific immune memory that enhances their responses to subsequent related or unrelated challenges, a phenomenon called “trained immunity” [42,43,44]. It was reported that trained alveolar macrophages induced by a primary respiratory viral infection are characterized by an enhanced capacity to produced cytokines upon restimulation, as well as by higher MHC-II expression [43]. Furthermore, it was demonstrated that IFN-γ has an important role in the generation of trained alveolar macrophages [42]. In line with these studies, we observed recently that the CD45^+^CD11c^+^SiglecF^+^ alveolar macrophages from infant mice treated nasally with NV1505 or PG1505 and infected with RSV had an enhanced capacity to produce IFN-γ, TNF-α, IL-6, CCL2, CXCL2 and CXCL10 in response to *S. pneumoniae* infection, as well as increased MHC-II expression. Thus, we speculated that nasally administered postimmunobiotics contribute to the generation of trained alveolar macrophages during RSV infection or TLR3 activation, in this way increasing the protection against secondary pneumococcal pneumonia [10,11,12,13,14]. Of note, it was shown that the protection against secondary challenges induced by trained immunity lasts for a long period: 1 to 4 months after the primary challenge [42,43,45]. In this work, then, we aimed to perform a more precise investigation of the duration of the immunomodulatory effects induced by NV1505 and PG1505 in the protection against secondary pneumococcal infection. In line with our hypothesis on the generation of trained alveolar macrophages, we observed that the protective effect of NV1505 and PG1505 against secondary *S. pneumoniae* infection persisted for 1 month. These results encourage us to carry out further studies, such as metabolic and epigenetic changes in NV1505- and PG1505-treated alveolar macrophages, to conclusively determine the induction of trained immunity.

## 5. Conclusions

This work advances in the characterization of the protective effect of NV1505 and PG1505 by demonstrating their ability to protect against the secondary infection produced by pneumococcal serotypes 3, 6B, 14 and 19F, with an effect that lasts up to 30 days after the primary viral inflammation. The results also confirm that the immunomodulatory properties of NV1505 and PG1505 are unique and are not shared by all the other members of this species, and suggest a capacity to stimulate trained immunity in alveolar macrophages. An important point for future studies is to find the bacterial molecules and immune receptors involved in the modulation of respiratory immunity by *L. rhamnosus*, which could explain the differences between the strains, particularly between NV1505 and NVIBL027. We demonstrated a distinct capacity of *L. plantarum* strains to regulate antiviral immunity in the intestine [24,25,26,27]. Our recent comparative genomic study showed a great variability in the predicted surface proteins of those *L. plantarum* strains, suggesting that the surface molecules could be involved in their differential ability to modulate the intestinal innate immune response against viruses [46]. A similar approach should be of value for *L. rhamnosus* in the context of respiratory immunity. Furthermore, detailed structural and composition studies of the different peptidoglycans could provide information to better understand the differences between the strains evaluated here.

## Data Availability

All the data related to this project are presented here.

## Supplementary Materials

The following supporting information can be downloaded at: https://www.mdpi.com/article/10.3390/microorganisms10112185/s1, Supplementary Figure S1. Ability of non-viable (NV) *L. rhamnosus* CRL1505, CRL498, CRL576, UCO25A and IBL027 and their peptidoglycan (PGs) to enhance resistance to secondary pneumococcal pneumonia after poly(I:C) treatment over time. Infant mice were nasally primed with NVs or PGs over two consecutive days. They were stimulated with three once-daily doses of poly(I:C) and finally challenged with *S. pneumoniae* on day 30 after the last poly(I:C) administration. Lung bacterial cells counts, hemocultures and albumin concentrations in bronchoalveolar lavages (BAL) as well as the levels of interferon (IFN)-β, IFN-γ and interleukin (IL)-10 in BAL were determined on day 2 post-pneumococcal challenge. The multiple comparisons of the magnitude of the fold expression changes with respect to the control are shown for the NV1505 vs. NVUCO25A vs. NVIBL027, NV1505 vs. NV489 vs. NV576, PG1505 vs. PGUCO25A vs. PGIBL027 and PG1505 vs. PG489 vs. PG576 groups. Supplementary Figure S2. Window of opportunity to improve the protection against the secondary pneumococcal pneumonia by non-viable (NVs) *L. rhamnosus* CRL1505, CRL498, CRL576, UCO25A and IBL027 and their peptidoglycan (PG) treatments. Infant mice were nasally treated with NVs or PGs 2 days after the administration of poly(I:C). *S. pneumoniae* infection was induced on days 5 after the last poly(I:C) administration. Lung bacterial cells counts, hemocultures and albumin concentrations in bronchoalveolar lavages (BAL) as well as (B) the levels of interferon (IFN)-β, IFN-γ and interleukin (IL)-10 BAL were determined on day 2 post-pneumococcal challenge. The multiple comparisons of the magnitude of the fold expression changes with respect to the control are shown for the NV1505 vs. NVUCO25A vs. NVIBL027, NV1505 vs. NV489 vs. NV576, PG1505 vs. PGUCO25A vs. PGIBL027 and PG1505 vs. PG489 vs. PG576 groups. Supplementary Figure S3. Ability of non-viable (NV) *L. rhamnosus* CRL1505, CRL498, CRL576, UCO25A and IBL027 and their peptidoglycan (PGs) to enhance resistance to secondary pneumococcal pneumonia produced by *S. pneumoniae* serotype 3. Infant mice were nasally primed with NVs or PGs for two consecutive days, then stimulated with three once-daily doses of poly(I:C) and finally challenged with S. pneumoniae serotype 3 five days after the last administration of poly(I:C). Non-treated infant mice stimulated with poly(I:C) and then challenged with the respective strain of *S. pneumoniae* were used as controls. Lung bacterial cells counts, hemocultures and albumin concentrations in bronchoalveolar lavages (BAL) as well as the levels of interferon (IFN)-β, IFN-γ and interleukin (IL)-10 in BAL were determined on day 2 post-pneumococcal challenge. The multiple comparisons of the magnitude of the fold expression changes with respect to the control are shown for the NV1505 vs. NVUCO25A vs. NVIBL027, NV1505 vs. NV489 vs. NV576, PG1505 vs. PGUCO25A vs. PGIBL027 and PG1505 vs. PG489 vs. PG576 groups.
