# Supplementary material for: The Ability of Postimmunobiotics from L. rhamnosus CRL1505 to Protect against Respiratory Syncytial Virus and Pneumococcal Super-Infection Is a Strain-Dependent Characteristic
Source: Microorganisms. 2022 Nov 3;10(11):2185. doi: 10.3390/microorganisms10112185 (PMC9694915; doi:10.3390/microorganisms10112185)
Supplement: Supplementary file 1 [file microorganisms-10-02185-s001.zip › microorganisms-1977852-supplementary.pdf]

microorganisms

MDPI

Supplementary Materials

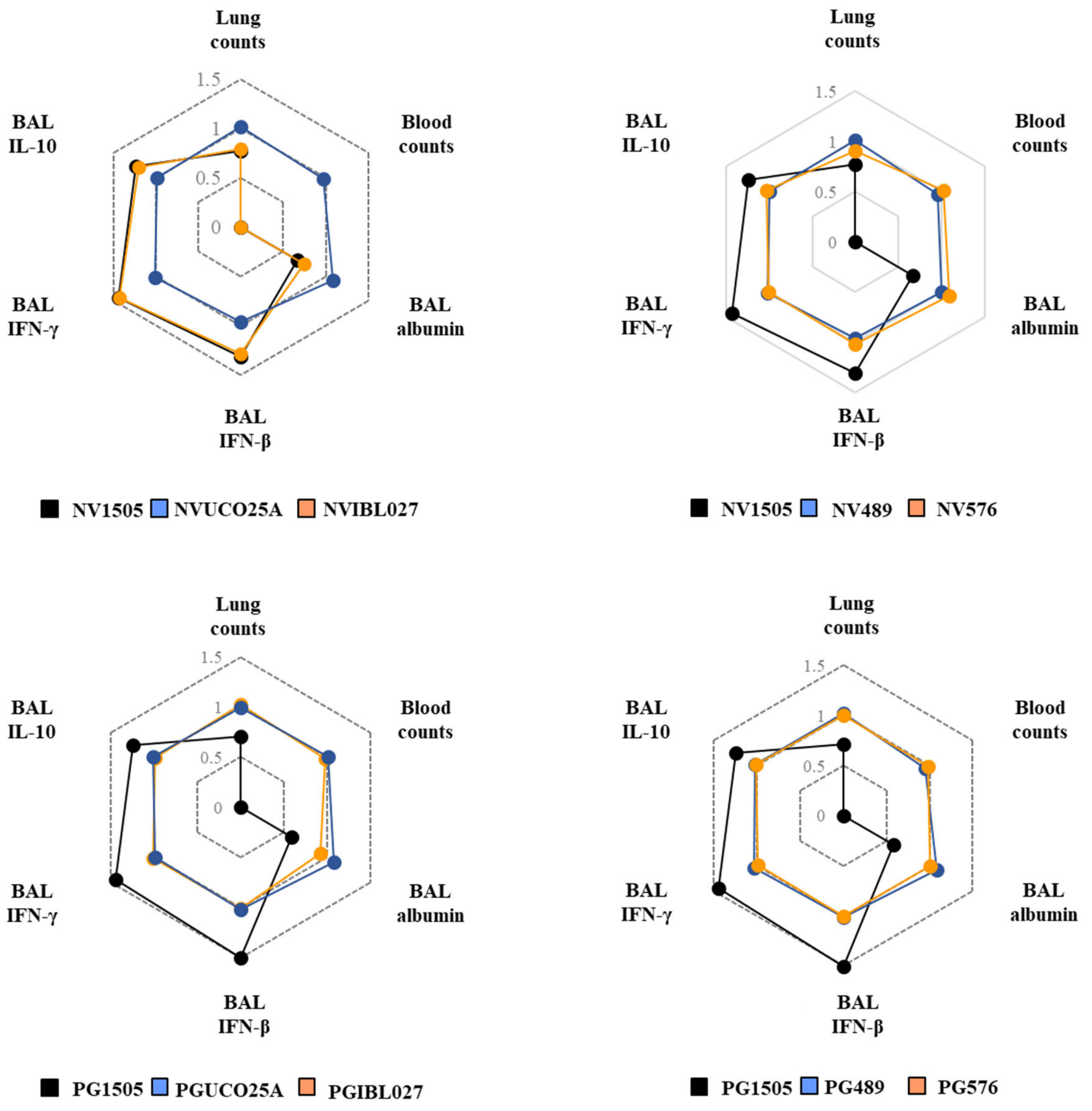

Figure S1. Ability of non-viable (NVs) *L. rhamnosus* CRL1505, CRL498, CRL576, UCO25A and IBL027 and their peptidoglycan (PGs) to enhance resistance to secondary pneumococcal pneumonia after poly(I:C) treatment over time. Infant mice were nasally primed with NVs or PGs during two consecutive days. They were stimulated with three once-daily doses of poly(I:C) and finally challenged with *S. pneumoniae* on day 30 after the last poly(I:C) administration. Lung bacterial cells counts, hemocultures and albumin concentrations in bronchoalveolar lavages (BAL) as well as the levels of interferon (IFN)-β, IFN-γ, and interleukin (IL)-10 in BAL were determined on day 2 post-pneumococcal challenge. The multiple comparisons of the magnitude of the fold expression changes with respect to the control are shown for the NV1505 vs. NVUCO25A vs. NVIBL027, NV1505 vs. NV489 vs. NV576, PG1505 vs. PGUCO25A vs. PGIBL027 and PG1505 vs. PG489 vs. PG576 groups.

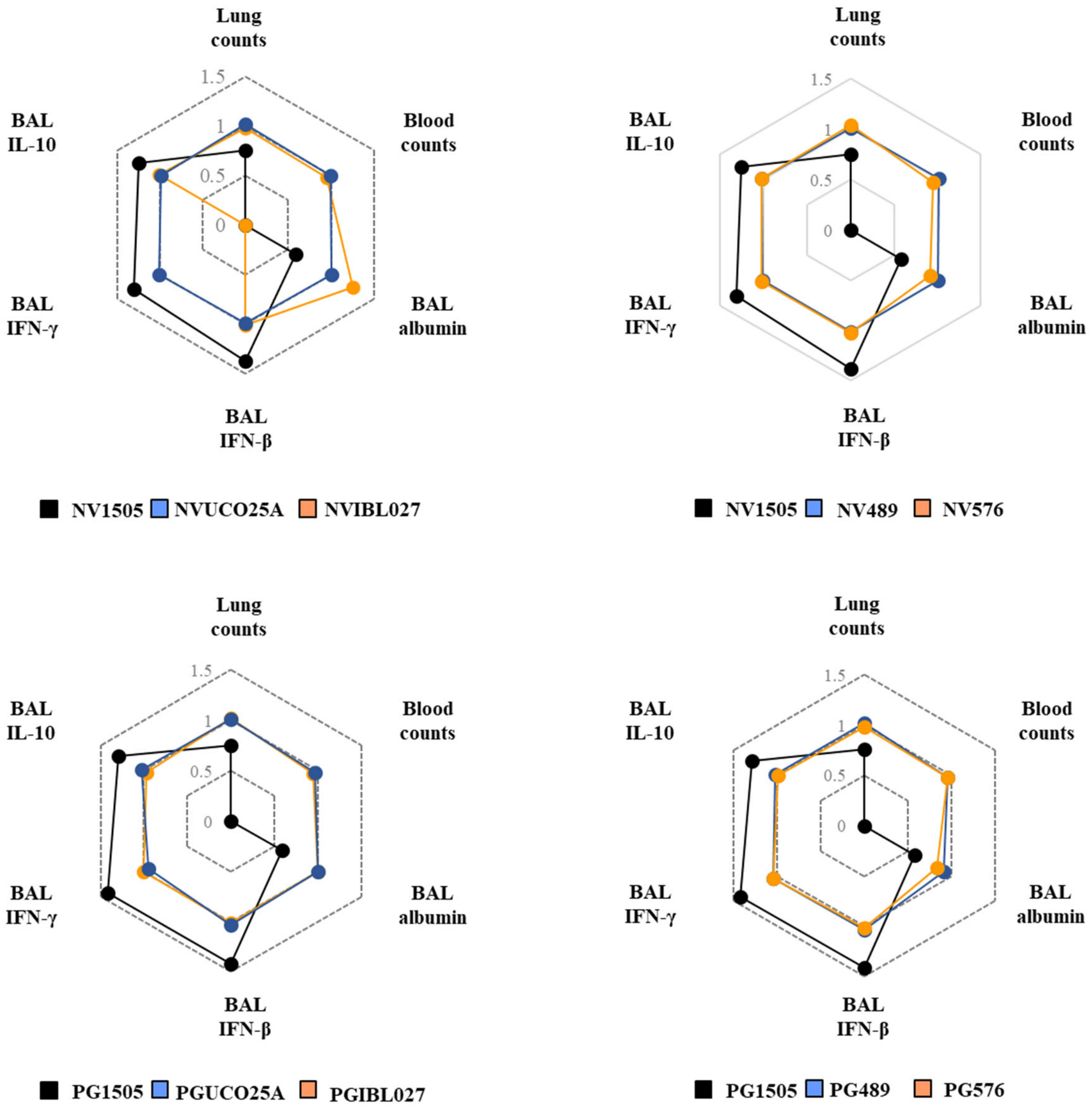

Figure S2. Window of opportunity to improve the protection against the secondary pneumococcal pneumonia by non-viable (NVs) *L. rhamnosus* CRL1505, CRL498, CRL576, UCO25A and IBL027 and their peptidoglycan (PGs) treatments. Infant mice were nasally treated with NVs or PGs 2 days after the administration of poly(I:C). *S. pneumoniae* infection was induced on days 5 after the last poly(I:C) administration. Lung bacterial cells counts, hemocultures and albumin concentrations in bronchoalveolar lavages (BAL) as well as (B) the levels of interferon (IFN)-β, IFN-γ, and interleukin (IL)-10 BAL were determined on day 2 post-pneumococcal challenge. The multiple comparisons of the magnitude of the fold expression changes with respect to the control are shown for the NV1505 vs. NVUCO25A vs. NVIBL027, NV1505 vs. NV489 vs. NV576, PG1505 vs. PGUCO25A vs. PGIBL027 and PG1505 vs. PG489 vs. PG576 groups.

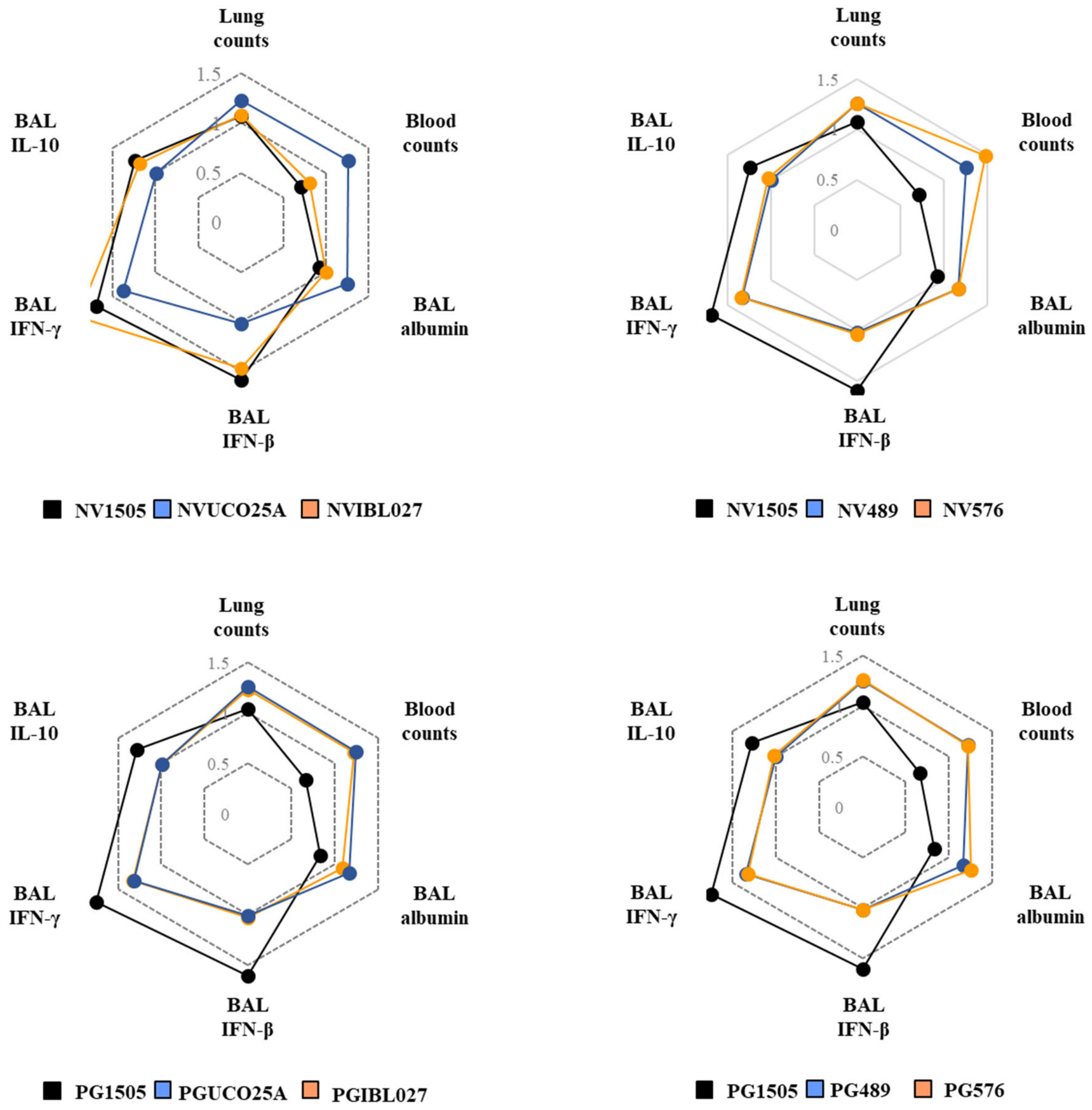

Figure S3. Ability of non-viable (NVs) *L. rhamnosus* CRL1505, CRL498, CRL576, UCO25A and IBL027 and their peptidoglycan (PGs) to enhance resistance to secondary pneumococcal pneumonia produced by *S. pneumoniae* serotype 3. Infant mice were nasally primed with NVs or PGs during two consecutive days, then stimulated with three once-daily doses of poly(I:C) and finally challenged with S. pneumoniae serotype 3 five days after the last administration of poly(I:C). Non-treated infant mice stimulated with poly(I:C) and then challenged with the respective strain of *S. pneumoniae* were used as controls. Lung bacterial cells counts, hemocultures and albumin concentrations in bronchoalveolar lavages (BAL) as well as the levels of interferon (IFN)-β, IFN-γ, and interleukin (IL)-10 in BAL were determined on day 2 post-pneumococcal challenge. The multiple comparisons of the magnitude of the fold expression changes with respect to the control are shown for the NV1505 vs. NVUCO25A vs. NVIBL027, NV1505 vs. NV489 vs. NV576, PG1505 vs. PGUCO25A vs. PGIBL027 and PG1505 vs. PG489 vs. PG576 groups.
